# PD-1 blockade and radiotherapy combination for advanced Epstein-Barr virus-associated intrahepatic cholangiocarcinoma: a case report and literature review

**DOI:** 10.3389/fimmu.2023.1239168

**Published:** 2023-09-11

**Authors:** Chun-Xu Liao, Chang-Song Deng, Xia Liang, Jian-Chuan Yang, Zhi-Zhong Chen, Xiao-Ying Lin, Cai-Feng Lin, Shen Chen, Song-Song Wu

**Affiliations:** ^1^ Department of Ultrasonography, Fujian Provincial Hospital, Shengli Clinical College of Fujian Medical University, Fuzhou, China; ^2^ Department of Ultrasonography, Affiliated Sanming First Hospital, Fujian Medical University, Sanming, China; ^3^ Department of Ultrasonography, Ningde Hospital, Ningde Hospital Affiliated to Ningde Normal University, Ningde, China; ^4^ Department of Pathology, Fujian Provincial Hospital, Shengli Clinical College of Fujian Medical University, Fuzhou, China

**Keywords:** Epstein-Barr virus, intrahepatic cholangiocarcinoma, immunotherapy, radiotherapy, complete remission

## Abstract

Advanced intrahepatic cholangiocarcinoma (ICC) is a rare malignant tumor of biliary epithelial cells, known for its extremely unfavorable prognosis. In the absence of intervention, patients typically survive for less than 5 months. Current guidelines from the Chinese Society of Clinical Oncology (CSCO), National Comprehensive Cancer Network (NCCN), and European Society for Medical Oncology (ESMO) recommend chemotherapy-based systemic therapy as the standard treatment for advanced ICC. However, the first-line regimen, consisting of gemcitabine in combination with cisplatin, generally results in a median survival of approximately one year, which is considered suboptimal. Significant progress has been made in radiotherapy techniques, molecular diagnostics, and tumor immune microenvironments. The integration of immune and radiation therapies has revolutionized treatment strategies for cholangiocarcinoma. Moreover, combined therapeutic regimens have shown promising results in improving survival rates among patients with advanced ICC. In this study, we present a case report of a 70-year-old male patient diagnosed with stage IV ICC, featuring metastases to the retroperitoneal, left adrenal, and left supraclavicular lymph nodes. The patient exhibited a high tumor mutational load, significant microsatellite instability, and hyper-expression of PD-L1 (90%), along with positive Epstein-Barr virus-encoded RNA (EBER). Pembrolizumab, a programmed cell death 1 (PD-1) inhibitor, was administered in conjunction with radiotherapy. As a result, considerable shrinkage and inactivation of the primary foci were observed, accompanied by the disappearance of metastases. Ultimately, the patient achieved complete remission and maintained progression-free survival for 41 months following the initial treatment. To the best of our knowledge, this represents the longest case of complete remission using a combination of immunotherapy and radiotherapy as a first-line regimen for the high tumor mutational load, microsatellite instability, and PD-L1 expression (90%) subtype of Epstein-Barr virus-associated ICC (EBVaICC). These findings suggest that the combination of PD-1 inhibitors with radiotherapy may serve as a promising therapeutic strategy for treating this particular cancer subtype.

## Introduction

Intrahepatic cholangiocarcinoma (ICC) represents the second most prevalent primary liver malignancy, accounting for approximately 10-15% of all primary liver cancers (1). In recent years, there has been a steady increase in the morbidity and mortality associated with ICC (2). Epstein-Barr virus (EBV)-associated ICC (EBVaICC) constitutes approximately 6.6% of all ICC cases (3). At the time of diagnosis, a significant proportion of patients (ranging from 60% to 88%) have already experienced local tumor progression or distant metastases, rendering surgical resection unfeasible and resulting in an overall unfavorable prognosis. Typically, patients with ICC survive for less than 5 months without intervention (4).

Current therapeutic options for cholangiocarcinoma include surgical resection, chemotherapy, radiotherapy, targeted therapy, immunotherapy, Transcatheter arterial chemoembolization (TACE), and combination therapy (5–7). Chemotherapy-based systemic therapy is recommended as an alternative treatment approach for patients with advanced ICC who are not suitable candidates for surgery. Currently, gemcitabine and cisplatin (GEMCIS) represent the preferred first-line therapy for advanced ICC according to existing guidelines (8, 9). However, despite its efficacy, patients receiving this treatment typically experience a median survival period of only one year (1). Nevertheless, significant progress in radiotherapy technology, molecular diagnostics, and the understanding of tumor immune microenvironments has undoubtedly revolutionized the care paradigm for cholangiocarcinoma. Combined therapy has demonstrated a substantial improvement in the survival rate for patients with advanced ICC (10). Furthermore, multiple studies have shown that radiotherapy enhances the therapeutic efficacy of immunotherapy by sensitizing the immune response (11). Nonetheless, the available evidence supporting the use of combined immune-radiotherapy as a first-line treatment strategy remains limited. This report presents a case of advanced EBVaICC with a high tumor mutation burden (TMB), microsatellite instability (MSI), and programmed death-ligand 1 (PD-L1) expression, which achieved long-term complete remission following the combination of radiotherapy with a PD-1 inhibitor (Pembrolizumab). The patient achieved a progression-free survival (PFS) of 41 months.

## Case presentation

**Figure 1:**
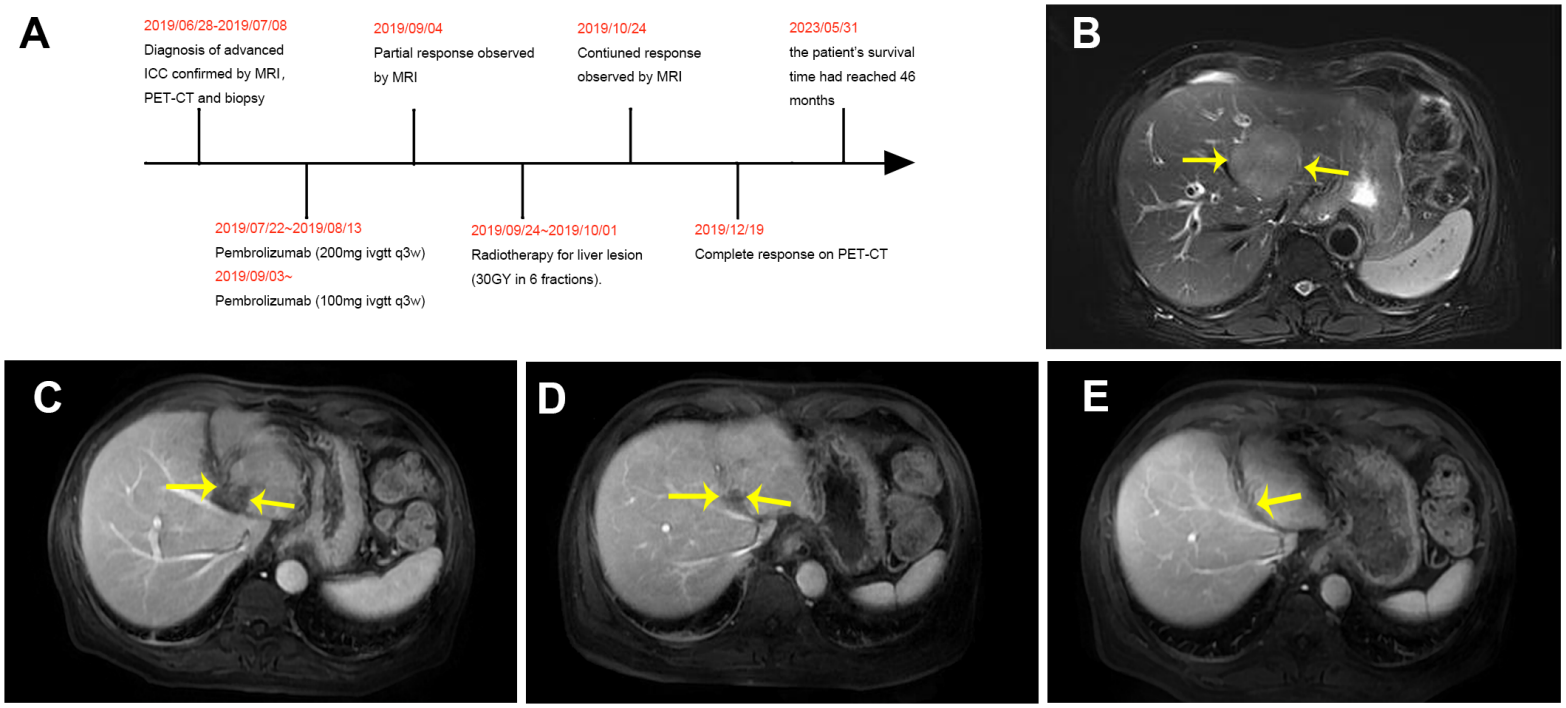
Timeline and MRI examination. Yellow arrows indicate the changes in lesion size. **(A)** Timeline of initial diagnosis, anti-PD-1 immunotherapy, radiotherapy and follow-up. **(B)** MRI results (June 28, 2019) prior to therapy. **(C)** MRI results (September 4, 2019) following 3 cycles of anti PD-1 treatment. **(D)** MRI results (October 24, 2019) following anti-PD-1 immunotherapy in combination with radiotherapy. **(E)** MRI results (April 8, 2020) following anti-PD-1 immunotherapy maintenance therapy.

**Figure 2:**
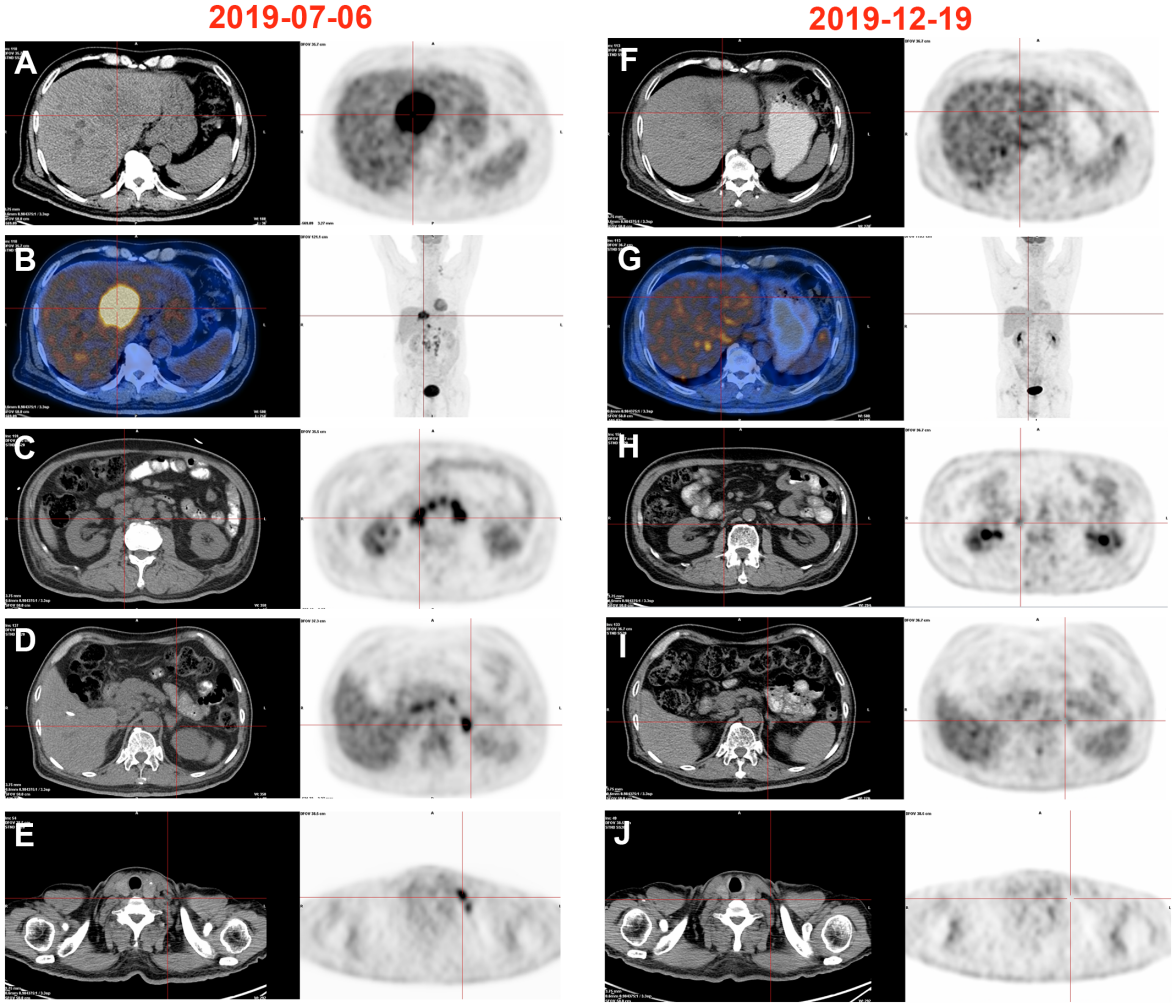
Changes of PET-CT images. **(A–E)** High metabolism in the left liver, retroperitoneum, left adrenal gland, and left supraclavicular lymphnode, 2019/07/06. **(F–J)** Hypermetabolism disappeared in the left liver, retroperitoneum, left adrenal gland, and left supraclavicular lymphnode, 2019/12/19.

A 70-year-old male patient presented with upper abdominal discomfort and jaundice was admitted to our hospital. Physical examination was unremarkable, and the patient had an Eastern Cooperative Oncology Group (ECOG) score of 1. Subsequent abdominal ultrasound imaging revealed a solid mass measuring approximately 69mm x 53mm in the S4 section of the liver, along with dilation of both left and right intrahepatic bile ducts. Enhanced computed tomography (CT) and magnetic resonance imaging (MRI) were performed for further diagnostic evaluation (**Figure 1B**), which supported a diagnosis of ICC. Laboratory tests showed elevated total bilirubin levels at 86.8 μmol/L, with direct bilirubin and indirect bilirubin levels of 41.6 μmol/L and 45.2 μmol/L, respectively. Tests for hepatitis B surface antigen, hepatitis C antibody, alpha-fetoprotein, and carcinoembryonic antigen (CEA) were negative, while blood test results for CA-19-9 indicated a value of 197.7 U/ml. A positron emission tomography-CT (PET-CT) examination (**Figures 2A–E**) revealed a hypermetabolic lesion in the left lobe of the liver, as well as enlarged and hypermetabolic lymph nodes in the upper retroperitoneum and left supraclavicular area, along with a hypermetabolic left adrenal gland. These findings collectively suggested cholangiocarcinoma in association with metastases to the lymph nodes and left adrenal gland. Following a multidisciplinary team (MDT) consultation, it was decided that the initial treatment stage would involve percutaneous transhepatic cholangial drainage (PTCD) to alleviate jaundice. On July 1, 2019, an ultrasound-guided PTCD intervention was performed on the right lobe of the liver. The jaundice index returned to normal levels within a week, enabling an ultrasonography-assisted guided puncture biopsy of the left lobe of the liver. Biopsy samples consisting of three strips of white intermixed flesh-colored tissue were collected using an 18G automatic biopsy device and sent for examination. Further analysis revealed medium to low differentiated cholangiocarcinoma, with an immunohistochemistry (IHC) profile of Ki67 (50% +), CK7 (++), CK20 (–), villin (++), CDX-2 (–), SATB-2 (–), CK19 (+), CEA (+), EMA (+++), Hep-Par-1 (–), Glypican-3 (+++), CD10 (–), CD117 (–), CD56 (–), ESA (+++), MOC31 (+++). According to the American Joint Committee on Cancer (AJCC) Cancer Staging Manual (8th edition), the patient was diagnosed with stage IV ICC (T1N1M1).

**Figure 3:**
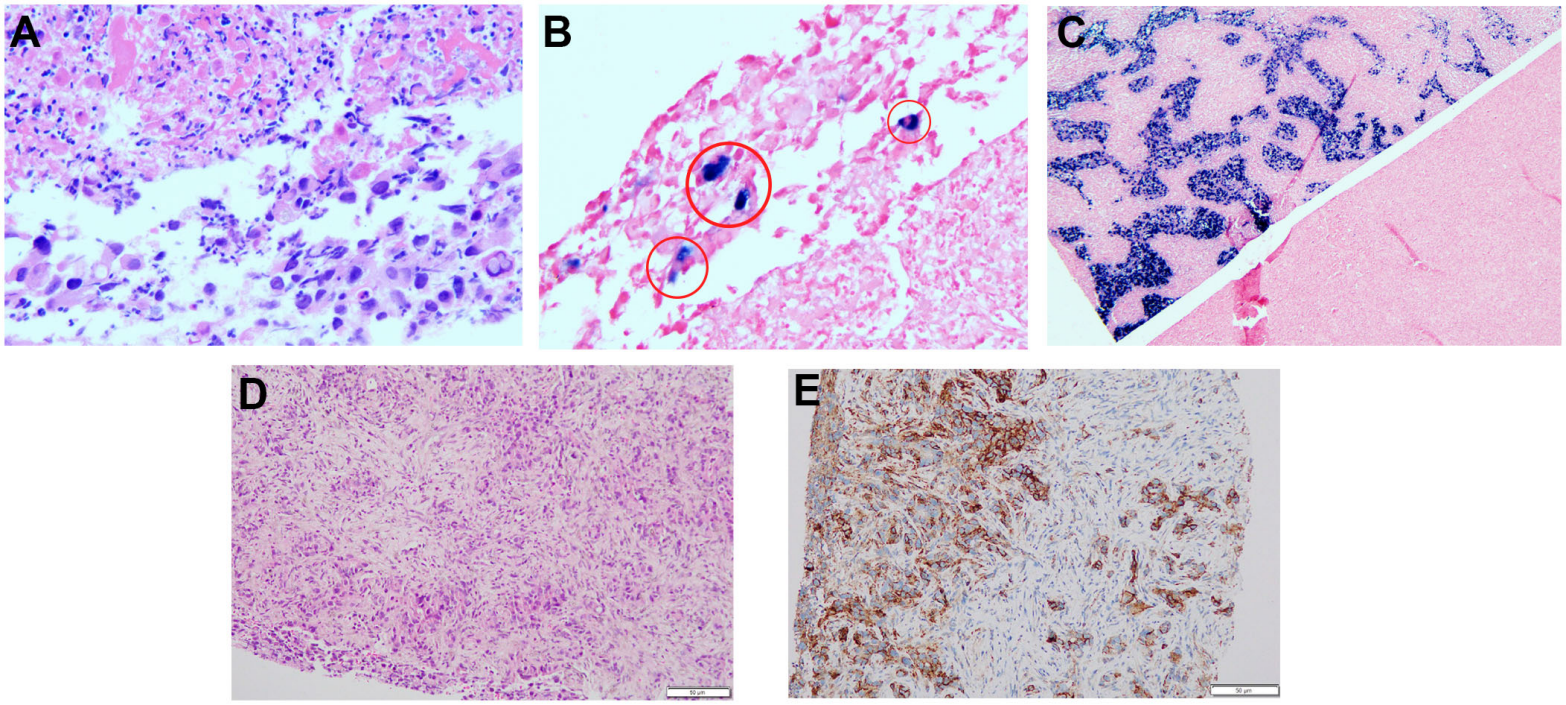
Hematoxylin-eosin HE staining and EBER, PD-L1 analysis of liver tumor tissues from the needle biopsy. **(A)** HE staining for EBER (200×). **(B)** EBER staining was positive in some areas, shown in red cicyle (200×). **(C)** EBER positive and negative comparison (50×). **(D)** HE staining for PD-L1. **(E)** Immunohistochemical staining for PD-L1 expression.

After obtaining informed consent from the patient and his family, a sequencing analysis of 1,021 genes was conducted on the biopsy samples obtained from the left hepatic mass to identify genetic variations associated with tumorigenesis. Additionally, the expression levels of Epstein-Barr virus-encoded RNA (EBER) and PD-L1 were examined. The genomic profiling revealed the presence of PTEN p. D268Gfs*30 (19.2%) and KRAS p.A146T (18.5%), along with high TMB (TMB-H, 53.76 Muts/Mb) and MSI (MSI-H). Immunohistochemistry analysis of PD-L1 exhibited a high tumor proportion score of 90% with staining observed on the tumor cell membrane. Furthermore, EBER was detected in the nuclei of specific liver tumor cells within the local area (**Figures 3A–E**). Subsequent to these test results, MDT consultation was conducted, and treatment with the PD-1 inhibitor Pembrolizumab was initiated on July 22, 2019, at a dosage of 200mg every three weeks. Targeted radiotherapy was administered to intrahepatic bile duct tumor lesions of the patient that were visible on imaging between September 24, 2019, and October 1, 2019, with a prescribed dose of gross tumor volume (GTV)-P 3000cGY/6f. Following targeted radiotherapy, an MRI conducted on October 24, 2019, demonstrated a significant reduction in the size of the liver tumor without enhancement, measuring approximately 38mm x 22mm (**Figures 1C–E**). A subsequent positron emission tomography/computed tomography (PET/CT) scan on December 19, 2019, revealed no hypermetabolism in the left liver lesion, retroperitoneal region, supraclavicular fossa, or left adrenal lesions, indicating no signs of disease progression in these areas (**Figures 2F–J**). Additionally, all tumor markers, including CA199, were within the normal range upon review. According to the criteria for evaluating the efficacy of solid tumors (version 1.1), the patient achieved complete remission. The patient continued Pembrolizumab maintenance therapy at a dosage of 100mg every three weeks without experiencing adverse events. Regular follow-up MRI scans were conducted every three months, consistently demonstrating regression in the size of the left liver lesion without enhancement and no dilation in the intrahepatic bile ducts. A follow-up PET-CT scan on July 7, 2021, showed no increase in the lesion or evidence of metastases. To date, there have been no signs of tumor recurrence or metastasis, as well as no reports of epigastric discomfort or jaundice. The patient’s appetite fully recovered, and his weight increased by 10 pounds. An illustrated timeline documenting the initial diagnosis, combined treatment, complete remission, and post-treatment follow-up is presented in **Figure 1A**.

## Discussion

The presence of advanced ICC often leads to local progression and distant organ metastasis, making surgical intervention impossible. Patients affected by this condition typically have an average survival time of less than 2 years, with only about 9% surviving beyond a 5-year timeframe (12). The current standard of care for treating advanced ICC involves the use of first-line systemic chemotherapy regimens like GEMCIS, as well as second-line options such as FOLFOX. Adjuvant systemic chemotherapy with capecitabine has also been demonstrated to be an effective treatment strategy for ICC patients. However, despite being the first-line treatment, GEMCIS has limited efficacy with a median PFS of only 8 months. Additionally, some patients cannot tolerate the significant toxicities associated with chemotherapy, including GEMCIS (13).Immunotherapy has brought about a paradigm shift in the established standard of care for various advanced and metastatic solid tumors, demonstrating the potential to achieve cures even in cases previously considered incurable (14). For advanced ICC, the TOPAZ-1 study revealed that a quarter of patients who received chemotherapy in combination with immunosuppression survived for more than 2 years (15). However, the majority of cholangiocarcinoma subtypes have a low response rate to immunotherapy, ranging from 3-22%, due to their immunodesert phenotype (16). Therefore, identifying ideal candidates for immunotherapy and selecting optimal combination therapy regimens remains challenging.

Recent advancements in etiological research, tumor immune microenvironment, and genetic diagnostics have paved the way for individualized and precise treatment approaches for patients with advanced ICC. EBVaICC, characterized by lymphoepithelioma-like histology, has shown a favorable response to immunotherapy, leading to improved survival rates (3). Within the liver, factors of tolerance such as PD-L1, expressed by Kupffer cells and dendritic cells, enable tumors to evade immune surveillance by upregulating PD-1 immune checkpoint and depleting T-cells (17). Blocking this pathway using PD-1/PD-L1 inhibitors can potentially restore T-cell activity, enhance the immune response, and counteract tumor recognition/attack by the immune system (18). Combining PD-1/PD-L1 inhibitors with histone deacetylase inhibitors has demonstrated an immunomodulatory effect (19). Moreover, genetic attributes such as TMB, MSI, and human leukocyte antigen class I (HLA-I) expression can impact tumor antigenicity and influence the response to immune-based therapy. TMB-H and MSI-H have been identified as reliable biomarkers for survival benefits related to immunotherapy for tumors (20–24). Moreover, Chowell et al. found that CD8 T cell-dependent tumor cell killing of tumor cells necessitates the efficient presentation of tumor antigens via HLA-I molecules. Additionally, maximal heterozygosity of HLA-I genes (A, B, C) has shown to improve overall survival in advanced cancer patients undergoing immune checkpoint blockade (25).

The relationship between immunotherapy and radiotherapy is complex. Radiation therapy can induce DNA damage, releasing tumor antigens and facilitating their recognition by antigen-presenting cells, thereby acting as an immunotherapeutic sensitizer (26). Combining radiotherapy with PD-1 blockade promotes T-cell infiltration and PD-L1 expression in tumor cells, enhancing the effectiveness of both treatments, especially in sensitizing local and distant metastatic lesions (27). External radiation radiotherapy has been established as a safe and effective treatment for advanced, unresectable ICC (10, 28–30). HON et al. reported that radiotherapy administered to patients with ICC resulted in a 2-year local control rate of 94.1%, and a 2-year survival rate of roughly 46.5% (28). Notably, combining anti-PD-1 immunotherapy with radiotherapy has shown a favorable response, as observed in this patient. The treatment resulted in complete inactivation of intrahepatic lesions and lymph node metastases, leading to long-term survival. **Table 1** provides a comprehensive literature review on the use of immunotherapy combined with radiation therapy for ICC treatment.

**Table 1:** Case reports summary of the ICC patients with Anti-PD-1 Immunotherapy and Radiotherapy.

Reference	Sex	Age	Type of tumor	AJCC stage	Molecular Biology and Genetic Testing	treatment	Clinical end point	Survival times(month)
LiuX2019 (27)	F	52	ICC	IV	TMB 1.2 Muts/Mb, pMMR and MSS, PD-L1 expression level < 1%.	1.SBRT+nivolumab, PR; 2.Maintenance therapy with apatinib and lenvatinib	Lesion diameter decreased by 40.9%	14
	M	59	ICC	IIIA (T3N0M0)	Amplification of ERBB2, TMB 3.8 muts/Mb, pMMR and MSS, PD-L1 expression level< 1%.	1.surgery+lapatinib (kinase inhibitor), PD; 2.pembrolizumab+SBRT, PR	Lesion diameters decreased by 86.3%	19
	M	51	ICC	IIIB (T2N1M0)	TMB 0.98 Muts/Mb, pMMR and MSS, PD-L1 expression level of < 1%.	1.surgery, recurrence 2.SBRT+pembrolizumab+chemotherapy +recombinant human endostatin, CR	PFS 11months	35
LiuZL2020 (31)	M	68	ICC	IV (T1N1M1)	TMB-H (16.9 Muts/Mb) and high MSI, low expression PD-L1, Low frequency of CD8+ T cells.	Pembrolizumab and radiotherapy, CR	/	26
ZhaoQ2021 (32)	M	50	ICC	IV (T2N1M1)	pMMR, TMB 7.5 mut/Mb, high numbers of PD-L1 positive tumor cells and abundant infiltration of CD4+ and CD8+ T cells.	1.SBRT,4 months after SBRT, PD; 2.Radiofrequency ablation (RFA) and chemotherapy, PD; 3.Nivolumab, 10 months later, CR	PFS 3months	21
	M	59	ICC	IB (T1bN0M0)	Weak positive expression of PD-L1, pMMR.	1.Surgery, PD; 2.Chemotherapy, PD; 3.RT+Nivolumab, SD	/	10
	M	52	ICC	IIIB (T4N1M0)	Few cells were positive for PD-L1, CD4, or CD8.	1.SBRT, PR; 2.Pembrolizumab+Everolimus, CR; 3.Surgery	PFS 3months	12
present case	M	70	ICC	IV (T1N1M1)	high MSI and TMB-H (53.76 Muts/Mb), PD-L1 expression level of 90%, EBER positive.	Pembrolizumab and radiotherapy, CR	PFS 41months	46

SBRT, stereotactic body radiotherapy; pMMR, proficient mismatch repair; CR, complete remission; SD, stable disease; PD, disease progression; PR, partial remission; PFS, progression-free survival; PD-L1, programmed cell death ligand 1; TMB, tumor mutation burden; MSI, microsatellite instability; Survival time, from diagnosis to the end of follow-up.

Selecting an appropriate treatment plan is crucial for effectively managing advanced ICC. One potential approach involves performing PTCD before biopsy to minimize bleeding and biliary leakage risks during the procedure. Thorough pathological assessment and genetic evaluations can help identify the most suitable treatment strategy and improve overall patient outcomes. This approach has significant implications for advancing tumor treatment models and may further catalyze the development of advanced tumor therapies.

## Conclusions

In this study, we administered a first-line treatment of anti-PD-1 immunotherapy combined with radiotherapy to a patient with advanced EBVaICC. Remarkably, the patient achieved long-term complete remission without experiencing significant toxicity and has now survived for 46 months. This outcome highlights the therapeutic potential of combining immunotherapy and radiotherapy as a treatment strategy for managing advanced EBVaICC patients who exhibit high levels of TMB, MSI, and PD-L1 expression. Moreover, our treatment regimen offers a unique and novel approach for the management of advanced ICC in this specific patient population.

## Data availability statement

The original contributions presented in the study are included in the article/**Supplementary Materials**, further inquiries can be directed to the corresponding author/s.

## Ethics statement

The studies involving humans were approved by ethics committee of Fujian Provincial Hospital. The studies were conducted in accordance with the local legislation and institutional requirements. The participants provided their written informed consent to participate in this study. Written informed consent was obtained from the individual(s) for the publication of any potentially identifiable images or data included in this article. Written informed consent was obtained from the participant/patient(s) for the publication of this case report.

## Author contributions

C-XL, C-SD, and XL contributed to the data collection. Z-ZC supervised the pathology interpretation of the patient tissue samples. C-XL and C-SD was responsible for writing the manuscript. J-CY and X-YL was responsible for the analysis of data. SC and S-SW was responsible for the interpretation and revision. All authors contributed to the article and approved the submitted version.

## References

[1] Moris D Palta M Kim C Allen PJ Morse MA Lidsky ME. Advances in the treatment of intrahepatic cholangiocarcinoma: An overview of the current and future therapeutic landscape for clinicians. CA Cancer J Clin (2023) 73:198–222. doi: 10.3322/caac.21759 36260350

[2] Bertuccio P Malvezzi M Carioli G Hashim D Boffetta P El-Serag HB. Global trends in mortality from intrahepatic and extrahepatic cholangiocarcinoma. J Hepatol (2019) 71:104–14. doi: 10.1016/j.jhep.2019.03.013 30910538

[3] Huang YH Zhang CZ Huang QS Yeong J Wang F Yang X. Clinicopathologic features, tumor immune microenvironment and genomic landscape of Epstein-Barr virus-associated intrahepatic cholangiocarcinoma. J Hepatol (2021) 74:838–49. doi: 10.1016/j.jhep.2020.10.037 33212090

[4] Shi GM Huang XY Wu D Sun HC Liang F Ji Y. Toripalimab combined with lenvatinib and GEMOX is a promising regimen as first-line treatment for advanced intrahepatic cholangiocarcinoma: a single-center, single-arm, phase 2 study. Signal Transduct Target Ther (2023) 8:106. doi: 10.1038/s41392-023-01317-7 36928584 PMC10020443

[5] Tsimafeyeu I Temper M. Cholangiocarcinoma: an emerging target for molecular therapy. Gastrointest Tumors. (2021) 8:153–8. doi: 10.1159/000517258 PMC8546446 34722468

[6] Li Y Song Y Liu S. The new insight of treatment in Cholangiocarcinoma. J Cancer. (2022) 13:450–64. doi: 10.7150/jca.68264 PMC8771522 35069894

[7] Zhang Y Esmail A Mazzaferro V Abdelrahim M. Newest therapies for cholangiocarcinoma: an updated overview of approved treatments with transplant oncology vision. Cancers (Basel). (2022) 14:5074. doi: 10.3390/cancers14205074 36291857 PMC9600404

[8] Vogel A Bridgewater J Edeline J Kelley RK Klümpen HJ Malka D. Biliary tract cancer: ESMO Clinical Practice Guideline for diagnosis, treatment and follow-up. Ann Oncol (2023) 34:127–40. doi: 10.1016/j.annonc.2022.10.506 36372281

[9] Benson AB D’Angelica MI Abbott DE Anaya DA Anders R Are C. Hepatobiliary cancers, version 2.2021, NCCN clinical practice guidelines in oncology. J Natl Compr Canc Netw (2021) 19:541–65. doi: 10.6004/jnccn.2021.0022 34030131

[10] Tao R Krishnan S Bhosale PR Javle MM Aloia TA Shroff RT. Ablative radiotherapy doses lead to a substantial prolongation of survival in patients with inoperable intrahepatic cholangiocarcinoma: A retrospective dose response analysis. J Clin Oncol (2016) 34:219–26. doi: 10.1200/JCO.2015.61.3778 PMC4980564 26503201

[11] Wang Y Liu ZG Yuan H Deng W Li J Huang Y. The reciprocity between radiotherapy and cancer immunotherapy. Clin Cancer Res (2019) 25:1709–17. doi: 10.1158/1078-0432.CCR-18-2581 PMC6420874 30413527

[12] Khan SA Tavolari S Brandi G. Cholangiocarcinoma: Epidemiology and risk factors. Liver Int (2019) 39 Suppl 1:19–31. doi: 10.1111/liv.14095 30851228

[13] Valle J Wasan H Palmer DH Cunningham D Anthoney A Maraveyas A. Cisplatin plus gemcitabine versus gemcitabine for biliary tract cancer. N Engl J Med (2010) 362:1273–81. doi: 10.1056/NEJMoa0908721 20375404

[14] SChadendorf D Hodi FS Robert C Weber JS Margolin K Hamid O. Pooled analysis of long-term survival data from phase II and phase III trials of ipilimumab in unresectable or metastatic melanoma. J Clin Oncol (2015) 33:1889–94. doi: 10.1200/JCO.2014.56.2736 PMC5089162 25667295

[15] Oh D He AR Qin S Chen L Okusaka T Vogel A. A phase 3 randomized, double-blind, placebo-controlled study of durvalumab in combination with gemcitabine plus cisplatin (GemCis) in patients (pts) with advanced biliary tract cancer (BTC): TOPAZ-1. J Of Clin Oncol (2022) 40:378–8. doi: 10.1200/JCO.2022.40.4_suppl.378

[16] Lin Y Peng L Dong L Liu D Ma J Lin J. Geospatial immune heterogeneity reflects the diverse tumor-immune interactions in intrahepatic cholangiocarcinoma. Cancer Discovery (2022) 12:2350–71. doi: 10.1158/2159-8290.CD-21-1640 35853232

[17] Heymann F Tacke F. Immunology in the liver–from homeostasis to disease. Nat Rev Gastroenterol Hepatol (2016) 13:88–110. doi: 10.1038/nrgastro.2015.200 26758786

[18] Kelley RK Bridgewater J Gores GJ Zhu AX. Systemic therapies for intrahepatic cholangiocarcinoma. J Hepatol (2020) 72:353–63. doi: 10.1016/j.jhep.2019.10.009 31954497

[19] Mazziotta C Lanzillotti C Gafà R Touzé A Durand MA Martini F. The role of histone post-translational modifications in merkel cell carcinoma. Front Oncol (2022) 12:832047. doi: 10.3389/fonc.2022.832047 35350569 PMC8957841

[20] Wang F Wei XL Wang FH Xu N Shen L Dai GH. Safety, efficacy and tumor mutational burden as a biomarker of overall survival benefit in chemo-refractory gastric cancer treated with toripalimab, a PD-1 antibody in phase Ib/II clinical trial NCT02915432. Ann Oncol (2019) 30:1479–86. doi: 10.1093/annonc/mdz197 PMC6771223 31236579

[21] Zhang Z Zhang W Wang H Hu B Wang Z Lu S. Successful treatment of advanced intrahepatic cholangiocarcinoma with a high tumor mutational burden and PD-L1 expression by PD-1 blockade combined with tyrosine kinase inhibitors: A case report. Front Immunol (2021) 12:744571. doi: 10.3389/fimmu.2021.744571 34603331 PMC8484748

[22] Diaz LA Jr Shiu KK Kim TW Jensen BV Jensen LH Punt C. Pembrolizumab versus chemotherapy for microsatellite instability-high or mismatch repair-deficient metastatic colorectal cancer (KEYNOTE-177): final analysis of a randomised, open-label, phase 3 study. Lancet Oncol (2022) 23:659–70. doi: 10.1016/S1470-2045(22)00197-8 PMC9533375 35427471

[23] Maio M Ascierto PA Manzyuk L Motola-Kuba D Penel N Cassier PA. Pembrolizumab in microsatellite instability high or mismatch repair deficient cancers: updated analysis from the phase II KEYNOTE-158 study. Ann Oncol (2022) 33:929–38. doi: 10.1016/j.annonc.2022.05.519 35680043

[24] Ricciuti B Wang X Alessi JV Rizvi H Mahadevan NR Li YY. Association of high tumor mutation burden in non-small cell lung cancers with increased immune infiltration and improved clinical outcomes of PD-L1 blockade across PD-L1 expression levels. JAMA Oncol (2022) 8:1160–8. doi: 10.1001/jamaoncol.2022.1981 PMC9204620 35708671

[25] Chowell D Morris L Grigg CM Weber JK Samstein RM Makarov V. Patient HLA class I genotype influences cancer response to checkpoint blockade immunotherapy. Science (2018) 359:582–7. doi: 10.1126/science.aao4572 PMC6057471 29217585

[26] Yi M Zheng X Niu M Zhu S Ge H Wu K. Combination strategies with PD-1/PD-L1 blockade: current advances and future directions. Mol Cancer. (2022) 21:28. doi: 10.1186/s12943-021-01489-2 35062949 PMC8780712

[27] Liu X Yao J Song L Zhang S Huang T Li Y. Local and abscopal responses in advanced intrahepatic cholangiocarcinoma with low TMB, MSS, pMMR and negative PD-L1 expression following combined therapy of SBRT with PD-1 blockade. J Immunother Cancer. (2019) 7:204. doi: 10.1186/s40425-019-0692-z 31383016 PMC6683483

[28] Hong TS Wo JY Yeap BY Ben-Josef E McDonnell EI Blaszkowsky LS. Multi-institutional phase II study of high-dose hypofractionated proton beam therapy in patients with localized, unresectable hepatocellular carcinoma and intrahepatic cholangiocarcinoma. J Clin Oncol (2016) 34:460–8. doi: 10.1200/JCO.2015.64.2710 PMC4872014 26668346

[29] Brunner TB Blanck O Lewitzki V Abbasi-Senger N Momm F Riesterer O. Stereotactic body radiotherapy dose and its impact on local control and overall survival of patients for locally advanced intrahepatic and extrahepatic cholangiocarcinoma. Radiother Oncol (2019) 132:42–7. doi: 10.1016/j.radonc.2018.11.015 30825968

[30] De B Tran Cao HS Vauthey JN Manzar GS Corrigan KL Raghav K. Ablative liver radiotherapy for unresected intrahepatic cholangiocarcinoma: Patterns of care and survival in the United States. Cancer (2022) 128:2529–39. doi: 10.1002/cncr.34223 PMC9177808 35417569

[31] Liu ZL Liu X Peng H Peng ZW Long JT Tang D. Anti-PD-1 immunotherapy and radiotherapy for stage IV intrahepatic cholangiocarcinoma: A case report. Front Med (Lausanne). (2020) 7:368. doi: 10.3389/fmed.2020.00368 32984358 PMC7485089

[32] Zhao Q Chen Y Du S Yang X Chen Y Ji Y. Integration of radiotherapy with anti-PD-1 antibody for the treatment of intrahepatic or hilar cholangiocarcinoma: reflection from four cases. Cancer Biol Ther (2021) 22:175–83. doi: 10.1080/15384047.2020.1834792 PMC8043185 33722163

